# Perceived stress in workers of Emergency Care Units in Palmas, Tocantins

**DOI:** 10.47626/1679-4435-2020-423

**Published:** 2020-12-11

**Authors:** Alyne Nunes Mota, Solange Maria Miranda Silva, Erika da Silva Maciel, Fernando Rodrigues Peixoto Quaresma

**Affiliations:** 1 Secretaria de Estado da Saúde do Tocantins (SES/TO) - Palmas (TO), Brazil; 2 Departamento de Enfermagem, Centro Universitário Luterano de Palmas - Palmas (TO), Brazil; 3 Departamento de Educação Física, Universidade Federal do Tocantins (UFT) - Miracema (TO), Brazil; 4 Departamento de Enfermagem, Universidade Federal do Tocantins (UFT) - Palmas (TO), Brazil

**Keywords:** stress, worker’s health, emergency medical services

## Abstract

**Background:**

Emergency Care Units are environments susceptible to occupational stress that can lead its staff members to illness.

**Objective:**

The objective of this study was to identify the levels of perceived stress in professionals who work in Emergency Care Units in the municipality of Palmas, state of Tocantins.

**Methods:**

This was a cross-sectional study conducted with 165 health care professionals working in Emergency Care Units. A structured questionnaire containing sociodemographic and economic aspects and the Perceived Stress Scale (EPS-10) were used.

**Results:**

The levels of perceived stress were identified in the 3 groups evaluated, and were more frequent among professionals with higher education, with an average of 17.8. There was a predominance of women (64.9%), 84.2% of the participants were public servants, 52.7% worked for longer than 12 hours, and 59.3% had an average income between R$ 790,00 and R$ 5.000,00.

**Conclusion:**

Professionals who work in the Emergency Care Units are under stress conditions. Health preventive and promotion measures should be developed and promoted to minimize this reality.

## INTRODUCTION

Access to health care is guaranteed free of charge for the Brazilian people, as provided in article 196 of the Brazilian Federal Constitution.^1^ Since the introduction of the Brazilian Unified Health System (SUS), the country has undergone several management models and changes that led to improvements in health care and, consequently, an increase in the demand for services.^2^ Despite the advances over the years, the complexity of the system and its characteristics, especially regarding its vast territorial area, the Brazilian Ministry of Health (MH) defined the public policy in the country based on thematic axes and interconnected by a Health Care Network (HCN).^2^^,^^3^ The restructuring of the network services provided the creation of the Urgent and Emergency Care Network (UECN) which, based on the Brazilian National Policy for Urgent Care, emerges as a proposal to strengthen the SUS guidelines, providing access to urgent and emergency services for all citizens.^4^

The UECN proposed by ordinance GM No. 1.600/2011 includes the Emergency Units as one of its components (UPAs 24 h -herein called only UPAs). The UPA “is the establishment of intermediate complexity health care between the Basic Health Units/ Family Health and the Hospital Network, which must compose an emergency care network.”^5^ It operates 24 hours a day, every day of the week, with the leading objective of welcoming patients, stabilizing their clinical condition, and counter-referring them to the network’s point of attention they need, with guaranteed access.^4^

Due to the operational characteristics of the UPA, the professionals who work in this environment are susceptible to stress. The factors intrinsic to stress, such as emotional exhaustion, dealing daily with tense work relationships and death, as well as extrinsic factors, such as an imbalance between work shifts, long hours, and inadequate conditions for performing work, were identified as conditioning factors for the illness in professionals who work in this setting.^6^^-^^9^

Stress represents a state produced by a change in the environment perceived as challenging, threatening or detrimental to a person’s balance, making individuals unable to meet the demands in the new situation. It is capable of directly influencing people’s lives and is considered one of the contemporary diseases because it directly interferes with the life of the individual, especially the health worker.^8^^-^^10^

Over the years, stress has been the object of several specialized scientific studies on health professionals, especially about the increase in occupational stress among these professionals, considered a public health problem,^11^ since it brings physical and psychological consequences to workers, and may trigger organic diseases or some significant dysfunction in the lives of professionals.^12^ The need to know the perception of stress among professionals who work in emergency services becomes essential to improve the quality of assistance to patients,^13^ justifying the importance of studies on the theme.

Thus, the objective of this study is to evaluate the perception of stress among health professionals who work in the UPAs in Palmas, state of Tocantins.

## METHOD

Cross-sectional study conducted according to the guidelines of the Strengthening the Reporting of Observational Studies in Epidemiology (STROBE),^14^ in 2 UPAs in the municipality of Palmas, one located in the southern region (150 professionals) and another in the northern region of the municipality (118 professionals), which received in 2013, the project Education through Work for Networks of Health Care - (PET-Health)/Urgent and Emergency Care of the MH. Data collection took place from November to December 2015. The approach followed the convenience sampling method and workers were invited to participate during their work activity. The Perceived Stress Scale (EPS-10) was administered, including 10 questions, in addition to a socioeconomic and demographic questionnaire.

The variables presented in the socioeconomic and demographic questionnaire were: sex, employment relationship, weekly work regime, years of service, number of work places, and total monthly income.

The perceived stress level was evaluated by the EPS-10, proposed by Cohen et al.^15^ and validated in Portuguese by Reis et al.^16^ The EPS-10 is composed of 10 multiple-choice questions aimed at assessing overall stress. For the answers, we considered the situations that were experienced in the previous month by the interviewee. The answers were rated on a Likert scale and the scores ranged from 0 to 40: the higher the score, the higher the perception of stress.

The data provided by the Municipal Health Department of Palmas estimated 268 professionals in both units, all potential participants. Thus, all professionals working in the UPAs agreed to sign a free and informed consent form (ICF) and were invited to participate voluntarily. However, only 165 professionals accepted to participate in this study and the percentage of loss was 38.4%.

The exclusion criteria for the research were to be either on leave or on vacation and to refuse or discontinue participation during the data collection process. The instruments were filled out according to face-to-face interviews conducted by a previously trained researcher.

The professionals were separated into 3 groups: higher education, technical, and secondary-level professionals. Among the professionals of higher-education level, the following professional categories were present: doctors (10), nurses (35), pharmacists (2), social service workers (4), and odontologists (4); among those of technical level: oral health technicians (4), nursing (62), X-ray (4), and laboratory (1); and among those of secondary level: administrative assistants (20), general services (10), security staff (5) and stretcher-bearers (4).

The data were entered into Epi Info 7.2^(®)^ (Centers for Disease Control and Prevention, Georgia, EUA). All data were validated in duplicate and when there was a discrepancy between pieces of information, a third researcher was consulted. The data obtained using the EPS-10 were tabulated and analyzed, and the general score was obtained with Microsoft Excel XP, 2007 (Microsoft Corporation, New Mexico, EUA). Descriptive statistics were also used (mean and standard deviation [SD]).

The collected data were processed and analyzed using SPSS 21.0 (IBM, New York, USA), according to the description of the data collection instruments, followed by descriptive analysis. The qualitative variables were described by absolute and relative frequencies, and quantitative variables, by mean, SD, and minimum and maximum values.

The project was submitted and approved by the Research Ethics Committee via Plataforma Brasil, according to resolution 466/2012,^17^ under approval number CAAE 39521014.7.0000.55160, and by the research evaluation committee of Foundational School of Public Health of Palmas (FESP), under number 055-11/2014.

This study was funded by PET-Health Urgent and Emergency Networks linked to the HM (Joint Ordinance No. 9, June 24, 2013).

## RESULTS

A total of 39 secondary-level professionals, 71 technical-level professionals, and 55 high-level professionals participated in the study, totaling 165 professionals among the categories who met the eligibility criteria.

Among the participants, there was a predominance of women among the professionals (64.9%), mainly in the technical and higher levels; 84.2% of the participants were public servants; 52.7% worked more than 12 hours on duty; 40.6% had been working in the units for less than 1 year; 46% worked in more than 2 locations, and 59.3% had a total monthly income between R$ 790.00 and R$ 5000.00 (Table 1).

**Table 1:** Socioeconomic and demographic characteristics of professionals in the Emergency Services Units (UPAs) of Palmas, state of Tocantins, 2015 (n = 165).

Variables	Mean (SD)/%		
	Secondary level	Technical level	Higher level
Sex			
Men	21 (53.8)	20 (28.2)	17 (30.9)
Women	18 (46.2)	51 (72.8)	38 (69.1)
Employment relation			
Public servant	30 (76.9)	59 (83.1)	50 (90.9)
Employee	9 (23.1)	10 (14.1)	5 (9.1)
Other	-	2 (2.8)	-
Work regimen (hours)			
6 to 8	1 (2.6)	5 (7.0)	7 (12.7)
10 to 12	20 (51.3)	32 (45.1)	13 (23.6)
More than 12	18 (46.2)	34 (47.9)	35 (63.6)
Time of experience (years)			
< 1	7 (17.9)	33 (46.5)	27 (49.1)
1 to 5	9 (23.1)	8 (11.3)	6 (10.9)
6 to 10	5 (12.8)	9 (12.7)	13 (23.6)
> 10	18 (46.2)	21 (29.6)	9 (16.4)
Number of places of work			
1	22 (56.4)	32 (45.1)	18 (32.7)
2	14 (35.9)	32 (45.1)	30 (54.5)
3	3 (7.7)	7 (9.9)	7 (12.7)
Total monthly income (in Brazilian R$)			
Not contributed	2 (5.1)	12 (16.9)	6 (10.9)
790.00 to 5000.00	32 (82.1)	52 (73.2)	14 (25.5)
6000.00 to 9000.00	5 (12.8)	7 (9.9)	19 (34.5)
10 000.00 to 19 000.00	-	-	11 (20.0)
> 20 000.00	-	-	5 (9.1)

SD: standard deviation.

Regarding the perceived levels of stress, we observed that the higher-level professionals had the highest mean stress, 17.8 (SD, 6.78), and the professionals who obtained the lowest mean were those with technical level, 15.46 (SD, 6.38) (Table 2).

**Table 2:** Perceived level of stress of higher-, technical-, and secondary-level professionals from the Emergency Care Units, (UPAs) in Palmas, state of Tocantins, 2015 (n = 165).

Variables	No	Minimum	Maximum	Mean ± SD
Secondary level	39	1	33	17.58±6.81
Technical level	71	2	33	15.46±6.38
Higher level	55	1	29	17.80±6.78

SD: standard deviation.

## DISCUSSION

In this article, we assessed the perception of stress among health professionals of secondary, technical, and higher levels who worked in the UPAs in the municipality of Palmas, state of Tocantins. The results of the study showed that there is a perception of stress in the 3 categories studied, with predominance among higher education professionals.

In relation to sex, the results are in line with studies on stress among health professionals: there is a predominance of women among the professionals studied.^18^^-^^20^ The current labor model includes a range of work relationships, and the instability of this relationship can directly impact the professional’s well-being.^21^ Although the employment relationship is indicative of a health problem for the worker, the precariousness of the employment relationship was not proven in this study, since 84.2% of the professionals who work in the UPAs are public servants.

Regarding the work regime of these professionals, it was observed that 52.7% of the professionals worked more than 12 hours a week, 40.6% had been working for less than 1 year in the UPAs, and 46% performed their activities in more than 1 workplace. Tito^22^ demonstrated that nurses have more than 1 job, confirming the data found in this study. In the UPAs, managers prefer to establish 12-hour shifts for professionals due to the 24-hour operation of the units.

With regard to the total monthly income for secondary-level and technical-level professionals, we observed that the wages, the number of employment contracts, and the exhaustive workload are situations that make technical professionals vulnerable and susceptible to stress.^23^ The perception of stress in this study proved to be more prevalent among higher-level education professionals (nurses, doctors, social workers, pharmacists, and dentists); several studies conducted with these professionals corroborated the results found in this study.^24^^-^^27^

In relation to the nurses, their high levels of stress may be due to work in the urgent and emergency services, the fact that they are multifaceted, work in an inadequate environment, suffer from a lack of materials and equipment to assist patients, constantly deal with the severity of cases, and often experience the death of patients.^28^^,^^29^ For physicians, stressors are related to long hours of work, less leisure time with family and friends, reduced rest time, sleep deprivation, charging the patient, more than one job, work overload, dissatisfaction, and pressure from the patient and family regarding treatment.^25^^,^^30^ The stress of social workers is due to the sometimes exaggerated burden of responsibility on these professionals and the role of establishing a connection between the patient and the state so that the treatment of this patient continues.^31^ Among pharmacists, there was also a high level of stress due to dissatisfaction with wages, excessive workload, and lack of recognition from the institution where they work.^24^ The perception of stress among dentists was also evidenced in a study by Marcelino Filho and Araújo.^32^

It is a challenge to change the reality of stress perception in the UPAs; however, it is necessary to consider actions that can promote the health of these professionals and seek management strategies so that they can perform their activities with commitment, dedication, and safety. Moreover, the most important is to ensure that they are motivated to have self-esteem and satisfaction in the work environment. It would be convenient to promote actions and training to reduce the levels of stress, so that professionals better deal with emergency and death situations. Promoting actions to improve the safety of professionals concerning the care provided to patients and their families would also be important, improving the quality of care and consequently reducing the level of stress of these professionals.

An important limitation was the difficulty in obtaining data from all professionals. However, the study was the first to assess the health of emergency staff members in the only 2 public units of intermediate complexity care in the capital of Tocantins, using instruments validated in Brazil, which constitutes an important parameter for similar studies.

These results should not be generalized because in the assessment of the levels of stress perceived by the workers of the emergency department we observed the presence of this risk factor among these professionals.

## CONCLUSIONS

This research aimed to assess the perception of stress among health professionals who work in the UPAs, and a higher prevalence was observed among health care professionals with higher education. Several factors are associated with the perception of stress by professionals working in urgent and emergency services. These factors directly impact the stress levels in these workers and, consequently, patient care.

In this sense, it is essential for managers to be aware of the levels of stress showed by these professionals, so that specific actions regarding worker health and quality of life are duly proposed to lessen or eliminate stress levels in both UPAs investigated.
